# Interactions between root endophytic microorganisms and the reduced negative ion release capacity of *Phalaenopsis aphrodite* Rchb. f. under high temperature stress

**DOI:** 10.3389/fpls.2024.1437769

**Published:** 2024-08-16

**Authors:** Qi Ye, Wenzhuo Lv, Yin Lu, Zili Wei, Yunxin Guo, Peijie Wang, Bingru Sun, Yumei Tong, Shenke Xuan, Wei Lin, Lijin Guo

**Affiliations:** ^1^ College of Life Sciences, Fujian Agriculture and Forestry University, Fuzhou, China; ^2^ College of Jun Cao Science and Ecology (College of Carbon Neutrality), Fujian Agriculture and Forestry University, Fuzhou, China; ^3^ International Magnesium Institute, College of Resources and Environment, Fujian Agriculture and Forestry University, Fuzhou, China; ^4^ College of Food Science, Fujian Agriculture and Forestry University, Fuzhou, China; ^5^ Fujian Agriculture and Forestry University (FAFU)-Dal Joint College, Fujian Agriculture and Forestry University, Fuzhou, China; ^6^ College of Economics and Management, Fujian Agriculture and Forestry University, Fuzhou, China; ^7^ College of Plant Protection, Fujian Agriculture and Forestry University, Fuzhou, China; ^8^ School of Foreign Languages, Guangzhou College of Technology and Business, Guangzhou, China; ^9^ College of Forestry, Fujian Agriculture and Forestry University, Fuzhou, China

**Keywords:** high temperature stress, *P. aphrodite*, negative oxygen ions, root endophytic microorganisms, peroxidase and proline content

## Abstract

**Introduction:**

Negative oxygen ions are produced by plants through photosynthesis, utilizing "tip discharge" or the photoelectric effect, which has various functions such as sterilization, dust removal, and delaying aging. With global warming, high temperatures may affect the ability of *Phalaenopsis aphrodite* Rchb. f. to produce negative oxygen ions. *P. aphrodite* is commonly used in modern landscape planning and forest greening.

**Methods:**

In this study, *P. aphrodite* was selected as the research object. By artificially simulating the climate, the control group (CK) and the high temperature stress group (HS) were set up in the experiment.

**Results:**

The study found that compared with the control group, the ability of *P. aphrodite* to produce negative oxygen ions significantly decreased when exposed to high temperature stress. Meanwhile, under high temperature stress treatment, peroxidase content increased by 102%, and proline content significantly increased by 35%.

**Discussion:**

Redundancy analysis results indicated a significant correlation between the root endophytic microbial community of *P. aphrodite* and negative oxygen ions, as well as physiological indicators. Under high temperature stress, *P. aphrodite* may affect the regulation of physiological indicators by modifying the composition of root endophytic microbial communities, thereby influencing the ability to release negative oxygen ions.

## 1 Introduction

Negative oxygen ions, also known as O_2_^-^(H_2_O)n, or OH^-^(H_2_O)n, or CO_4_(H_2_O)_2_, are a collection of negatively charged single gas molecules and light ion clusters (Yi et al., 2022). Under specific external conditions, gas molecules that are originally electrically neutral undergo changes, with the outermost electrons breaking free from the atomic nucleus and being released from their orbits, resulting in some gas molecules carrying positive charges. These positively charged gas molecules, along with the released electrons, combine with some neutral molecules or atoms to form cations or anions. The generation of negative oxygen ions is influenced by a series of macro-environmental factors and micro-biochemical reactions, such as various radioactive substances existing on the Earth’s lithosphere surface, causing water molecules in the air or water to split into positive and negative oxygen ions, leading to the combination of negative oxygen ions in the air with oxygen ions to form negative oxygen ions; materials with special structures and static electricity can ionize water molecules in the air to form negative oxygen ions; the phenomenon of “tip discharge” appearing at the pointed structures of trees in forests, such as tree leaves and tree canopy tips, and the photoelectric effect produced by plant photosynthesis (Shi et al., 2022), that is, the chlorophyll in the special state of photosynthesis in the process is stimulated and loses electrons when receiving light, and the current or voltage generated ionizes the air to produce negative oxygen ions; in addition, various radiation rays can also produce negative oxygen ions.

Negative oxygen ions have various functions such as disinfection and sterilization, purification and dust removal, prevention of bacterial spread, and enhancement of immune function (Shepherd et al., 2010; Jiang et al., 2018), playing a crucial role in environmental protection, human health, and delaying human aging (Chen et al., 2023). However, the occurrence of high-temperature stress can easily destroy the activity of plant proteins and mobile membrane lipids, affect the activity of chloroplast and mitochondrial enzymes and membrane integrity, lead to plant damage or death (Hu et al., 2020), and reduce the ability of plants to release negative oxygen ions. Therefore, it is significant to study the effect of high-temperature stress on the release of negative oxygen ions by plants to reveal the variation of negative oxygen ions concentration.

Currently, domestic and international research has focused on the effects of meteorological factors and plant communities on the variation of negative oxygen ions concentration but ignore the impact of environmental factors such as high temperature stress on the release capacity of negative oxygen ions by plant individuals. The changes of negative oxygen ions concentration in a high temperature environment are closely related to the vegetation photosynthesis process (Shi et al., 2022). High temperature significantly inhibits the generation of chlorophyll and its intermediates, reducing the ability of plants to produce negative oxygen ions (Kimm et al., 2021; Shi et al., 2022). However, the current studies are limited to the finding that high temperature can reduce the concentration of negative oxygen ions in the air, but the mechanism of the effect of high temperature stress on the production of negative oxygen ions in plants is rarely reported.

Rhizosphere microorganisms directly influence plant activity, enhancing plants’ ability to absorb nutrients and resist pathogens (Bachmann and Kinzel, 1992; Trivedi et al., 2020). The interaction between root endophytic microorganisms and plants can improve plants’ tolerance to stress, such as the heat-tolerant *Curvularia. protuberata* isolated from geothermal parasitic plants (De Zelicourt et al., 2013; Oleńska et al., 2020). Furthermore, the diversity of root endophytic microorganisms contributes to enhancing plants’ stress resistance, and increased root endophytic microorganisms diversity can promote plant growth and yield (Das et al., 2021; Hao et al., 2023). Additionally, factors such as plant genotype, growth environment, growth stage, and season can affect the structure and diversity of root endophytic microorganisms in plants (Jin et al., 2018). The secretions of root endophytic microorganisms can alter the physicochemical properties of the soil, thereby adjusting the community structure of microorganisms attached to the roots (Ai et al., 2023). The activity of root endophytic microorganisms is closely related to plant growth, and research on the relationship between root endophytic microorganisms in plants under high temperature stress and the plants’ ability to produce negative oxygen ions is still shallow, with the specific mechanisms awaiting further investigation.

To investigate the effect of high temperature stress on the ability of plants to produce negative oxygen ions, *Phalaenopsis aphrodite* Rchb. f. was selected as the research subject. *P. aphrodite* is a perennial herb of *Phalaenopsis* genus in the orchid family, known for its large and beautiful flowers, long flowering period, resistance to pests and diseases, and its usual growth in trees, not taking up natural space. It is commonly used in modern landscape planning, air purification, and forest greening. Through indoor potted plant experiments, the influence of high temperature stress on moth orchid physiological indicators, negative oxygen ions release capacity, and root endophytic microorganisms was observed, elucidating the microbial regulatory mechanism of high temperature stress on the negative oxygen ions production capacity of *P. aphrodite.*

## 2 Materials and methods

### 2.1 Experimental materials

The selected material for this experiment, *P. aphrodite*, was purchased from Fujian Yangta Horticulture Co., Ltd. Healthy and uniform 1.5-year-old seedlings were selected for the experiment and planted in plastic pots with a diameter of 2.7 inches. The substrate consisted of sphagna, fermented pine bark, corn cob, and sawdust. Each pot contained one *P. aphrodite*, placed under the following conditions: 14 h/10 h day/night, light intensity was the same as in the above of experiment as 37 μmol/m^2^/s, and indoor cultivation with a relative humidity of 70% to 80% for one month before subjecting them to high temperature stress.

### 2.2 Experimental design

The experiment was conducted at Miaofengshan Experimental Base of Fujian Agriculture and Forestry University in Cangshan District, Fuzhou City, Fujian Province (119.24744E, 26.07998N). High temperature stress was carried out in an artificial climate chamber (Lin et al., 2023), with the control group set at day/night (12 h/12 h) 25°C/20°C (CK, control group), and the high temperature stress treatment was day/night (12 h/12 h) 40°C/35°C (HS). Each treatment had 5 repetitions, with 6 pots of *P. aphrodite* per repetition. The other environmental conditions and management measures for each experimental group during the experiment were kept consistent with the control group, and the basic planting conditions were referred to the standardized cultivation specifications for *P. aphrodite*

After 3 days of high temperature stress, the concentration of negative oxygen ions was measured at regular intervals within 1 day for 12 hours, every two hours as a time period, a total of 6 times, and then the treated and control plants were immediately taken out, and the leaves and roots of *P. aphrodite* were harvested. The intact functional leaves of *P. aphrodite* with the top facing downwards were selected for physiological indicators measurement, and the collection of overly tender leaves was avoided. After collecting the plant leaf samples, *P. aphrodite* were completely removed from the flower pots, gently shaking the roots to reduce root hair breakage, removing most of the scattered soil from the roots, and gently brushing off the soil tightly connected to the roots, then rinsing the roots in clean water, draining them, placing them in sterile bags for detection and analysis of root endophytic microorganisms.

### 2.3 Index measurement and methods

#### 2.3.1 Measurement of negative oxygen ions

The HT-80 atmospheric ion monitor (Manufacturer: Shenzhen Hongtai Environmental Protection Technology Co., Ltd.) was selected for the experiment, with a measuring range of 1~1.999×10^9^ ion cm^-3^. In order to eliminate the influence of external environment on the measurement of negative oxygen ions (such as wind and the resulting changes in temperature and humidity), the measurement of the concentration of plant negative oxygen ions was conducted in a test box made of 4 mm thick organic glass with dimensions of 800 mm×800 mm×800 mm, which had great heat preservation, moisturization and airtightness. Each day was divided into 5 time periods from 9:00 to 21:00, and the start time was randomly selected in each time period. The negative oxygen ions concentration in the air was recorded every 20 seconds, 20 times in a row, and the average value for each time period was taken.

#### 2.3.2 Measurement of plant physiological indexes

Malondialdehyde content (MDA), proline content (Pro), soluble sugar content, soluble protein content, Superoxide dismutase (SOD), Catalase, POD (Peroxidase), APX (Ascorbate peroxidase) and Chl (Chlorophyl) were determined using the Solarbio kit (Beijing Solarbio Technology Co., LTD.), with specific methods following the instructions of the kits. The calculation formula is: Proline (Pro) content (μg/g fresh weight) =41.03×(ΔA+0.0064)÷W (ΔA-absorbance value, W-samplemass, g). A UV-vis spectrophotometer (UV-5500, Shanghai Meta Analysis) was used for measurement. All indexes were measured three times, and the average value was taken.

#### 2.3.3 Root endophytic microorganisms community sequencing

Root endophytic microorganisms community sequencing was conducted by Shanghai Paisenno Biological Technology Co., Ltd., using the Illumina Novaseq sequencing platform to amplify and construct libraries for the ITS gene ITS1-ITS2 region of fungi and the V5-V7 hypervariable region of the bacterial 16S rRNA gene. The fungal sequencing primers were ITS1F (5’-TCCGTAGGTGAACCTGCGC-3’) and ITS2R (5’-CTCGGACGAGGATCCTCGCC-3’) (Lin et al., 2022). All PCR reactions were performed in a 30 µL reaction mixture, which included 15 µL of Phusion^®^ High-Fidelity PCR Master Mix (New England Biolabs), 0.5 units of AccuPrimer TM Taq DNA Polymerase (Life Technologies, USA), 0.2 µM of forward and reverse primers, and 10 ng of template DNA. The thermal cycling conditions were as follows: initial denaturation at 98°C for 1 min, 30 cycles (98°C denaturation for 10 s, 50°C annealing for 30 s, 72°C extension for 60 s), and a final extension at 72°C for 5 min. The bacterial sequencing primers were 799F (5’-AACMGGATTAGTAGATACCCKG-3’) and 1193R (5’-ACGTCATCCCCACCTTCC-3’) (Shin et al., 2019), with specific primers including a barcode. The PCR reaction mixture (20 µL) comprised 2 µL of 10× PCR Buffer, 2 µL of 2.5 mmol L^-1^ dNTPs, 0.8 µL of 5 µmol L^-1^ forward primer, 0.8 µL of 5 µmol L^-1^ reverse primer, 0.2 µL of rTaq Polymerase, 0.2 µL of BSA, 10 ng of template DNA, and supplemented with ddH_2_O to 20 µL. The reaction conditions included 95 °C for 3 min; The reaction conditions included 95°Cfor 3 min; the number of cycles (95°C 30 s, 55°C 30 s, 72°C 45 s); 72°C for 10 min.

### 2.4 Data analysis

The negative oxygen ions concentration data were grouped according to the time period, and the concentration change line chart was drawn by Origin 2021. The plant physiological index data were calculated by the mean ± standard deviation (SD), and one-way analysis of variance was performed by R. The Duncan method was used for significance analysis, and the Paisenno Cloud platform (Shanghai Paisenno Biotechnology Co., Ltd.) was used for image drawing. The Alpha diversity index of endophytic microbial communities in the root system was calculated using the Vegan, Ggplot2, and Ggrepel packages in R. Redundancy analysis was performed using the R package (version 2.4.4 Date of access: October 11, 2023), and linear discriminant analysis and group difference analysis (LEfSe) were used for statistical analysis of microbial communities with significant differences between all treatment groups.

## 3 Results and analysis

### 3.1 The impact of high temperature stress on the negative oxygens ions production ability of *P. aphrodite*

**Figure 1:**
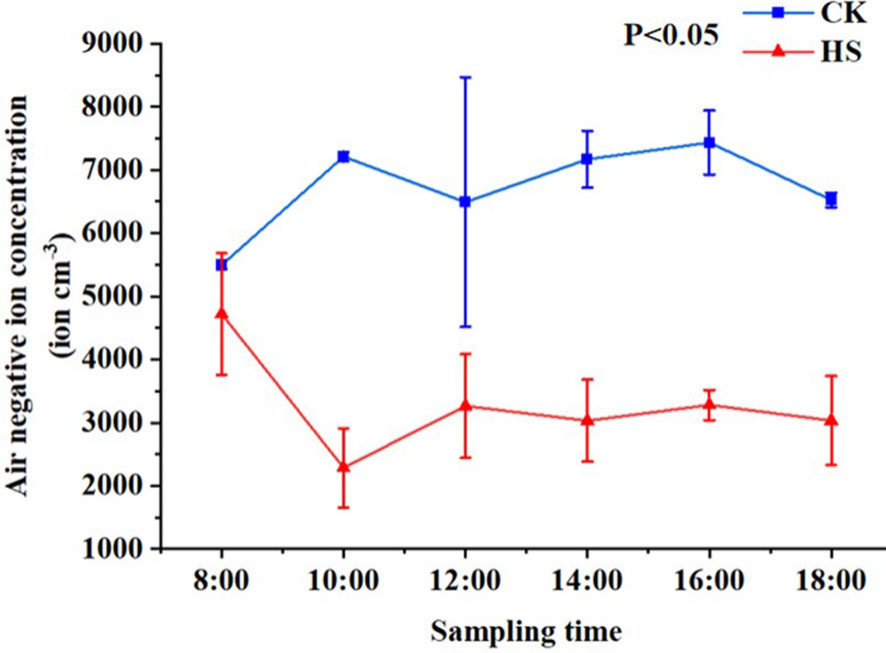
The changes in negative oxygen ions concentration. CK, control group; HS, high temperature stress treatment.

As shown in **Figure 1**. Under CK, the average negative oxygen ions concentration of times produced by *P. aphrodite* was higher than 5500 cm^-3^, while the average negative oxygen ions concentration was lower than 5000 cm^-3^ under high temperature stress treatment. Under normal conditions, the negative oxygen ions can peak at 10:00, then have large fluctuations, and decrease after 16:00. Under the condition of high temperature stress treatment, the concentration of negative oxygen ions produced by *P. aphrodite* was lower than that of CK treatment, and the concentration gradually decreased with time and reached the lowest value at 10:00, which recovered slightly since then, but fluctuated at a low level, indicating that high temperature stress would reduce the ability of *P. aphrodite* to produce negative oxygen ions.

### 3.2 Effects of high temperature stress on physiological indicators of *P. aphrodite*

**Figure 2:**
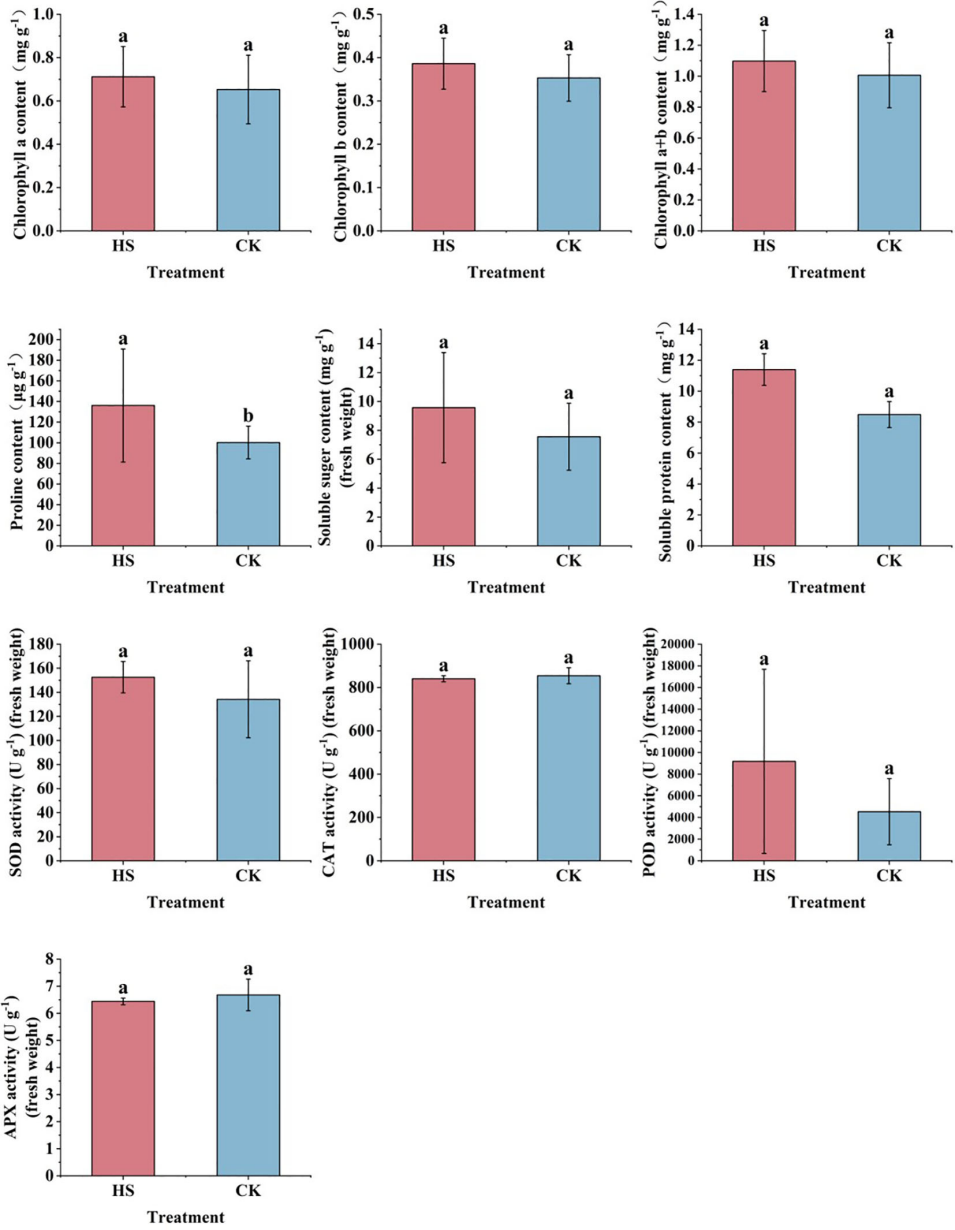
Changes in physiological indicators of *P. aphrodite* under different temperature treatments (6 days). CK, control group; HS, high temperature stress treatment; SOD, Superoxide dismutase; CAT, Catalase; POD, Peroxidase; APX, Ascorbate peroxidase; all values are expressed as mean ± standard deviation (n=5). Different lowercase letters indicate significant differences.

As shown in **Figure 2**, compared with CK, chlorophyll a, chlorophyll b, chlorophyll (a+b), soluble sugar, and soluble protein content are not significant after high temperature stress (P>0.05). The Pro content increased significantly by about 35% (P<0.05).

### 3.3 Effects of high temperature stress on the endophytic bacterial community in the root system of *P. aphrodite*

**Figure 3:**
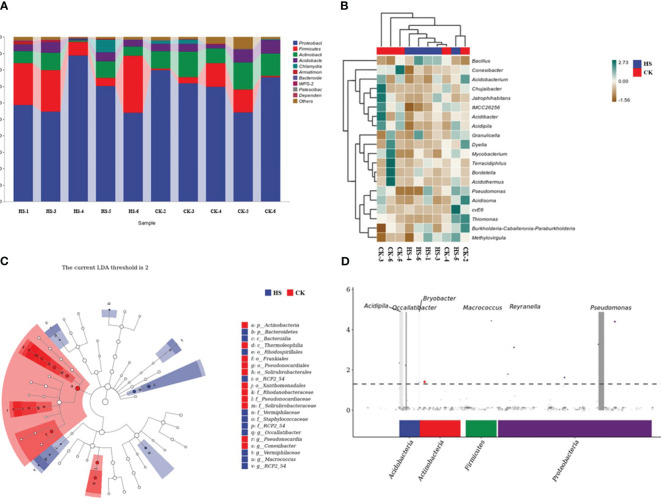
Occallatibacter, Pseudonocardia, Conexibacter, Vermiphilaceae, Macrococccus RCP2_54Comparison of endophytic bacterial community structure in *P. aphrodite* roots under high temperature stress conditions. **(A)** Analysis of bacterial taxonomic composition. **(B)** Heat map of bacterial species composition. **(C)** Bacterial LEfSe analysis. **(D)** Bacterial MetagenomeSeq analysis chart. CK, control group; HS, high temperature stress; p: phylum, o: order, c: class, f: family, g: genus. The data for **Figure 3A** are shown in **Supplementary Table S1**.

As shown in **Figure 3A**, at the genus level, *Proteobacteria* (67.7%), *Firmicutes* (13.2%), and *Firmicutes* (13.2%) were the top five endophytic bacteria in the root of *P. aphrodite*. *Actinobacteria* (9.9%), *Acidobacteria* (4.8%) and Chlamydiae (1.3%) accounted for a relatively small proportion of bacteria in other genera. Compared with CK, the relative abundance of *Proteobacteria* decreased by 7.1%, that of *Firmicutes* increased by 191.7%, that of *Actinobacteria* decreased by 47.3%, and that of *Acidobacteria* decreased by 25.5% under HS treatment. The relative abundance of Chlamydiae was increased by 77.3%.

**Figure 3B** shows that the abundance of Bacillus community was significantly increased under HS treatment compared with CK, while the abundance of *Conexibacter*, *Chujaibacter*, and *Dyella* was significantly decreased.

As shown in **Figure 3C**, under high temperature stress, *P. aphrodite* root endophytic bacteria, including Occallatibacter, Pseudonocardia, Conexibacter, Vermiphilaceae, Macrococccus, and RCP 2 _ 54, were significantly enriched at the genus level.

As shown in **Figure 3D**, at the phylum level, four phyla, Acidobacteria, Actinobacteria, Firmicutes and Proteobacteria, were significantly up-regulated under HS condition. At the genus level, the significantly up-regulated bacteria in Phalaenopsis roots under HS conditions were *Acidipila*, *Occallatibacter*, *Bryobacter*, *Macrococcus*, *Reyranella* and *Pseudomonas*.

### 3.4 Effects of high temperature stress on the endophytic fungal community in *P. aphrodite* root

**Figure 4:**
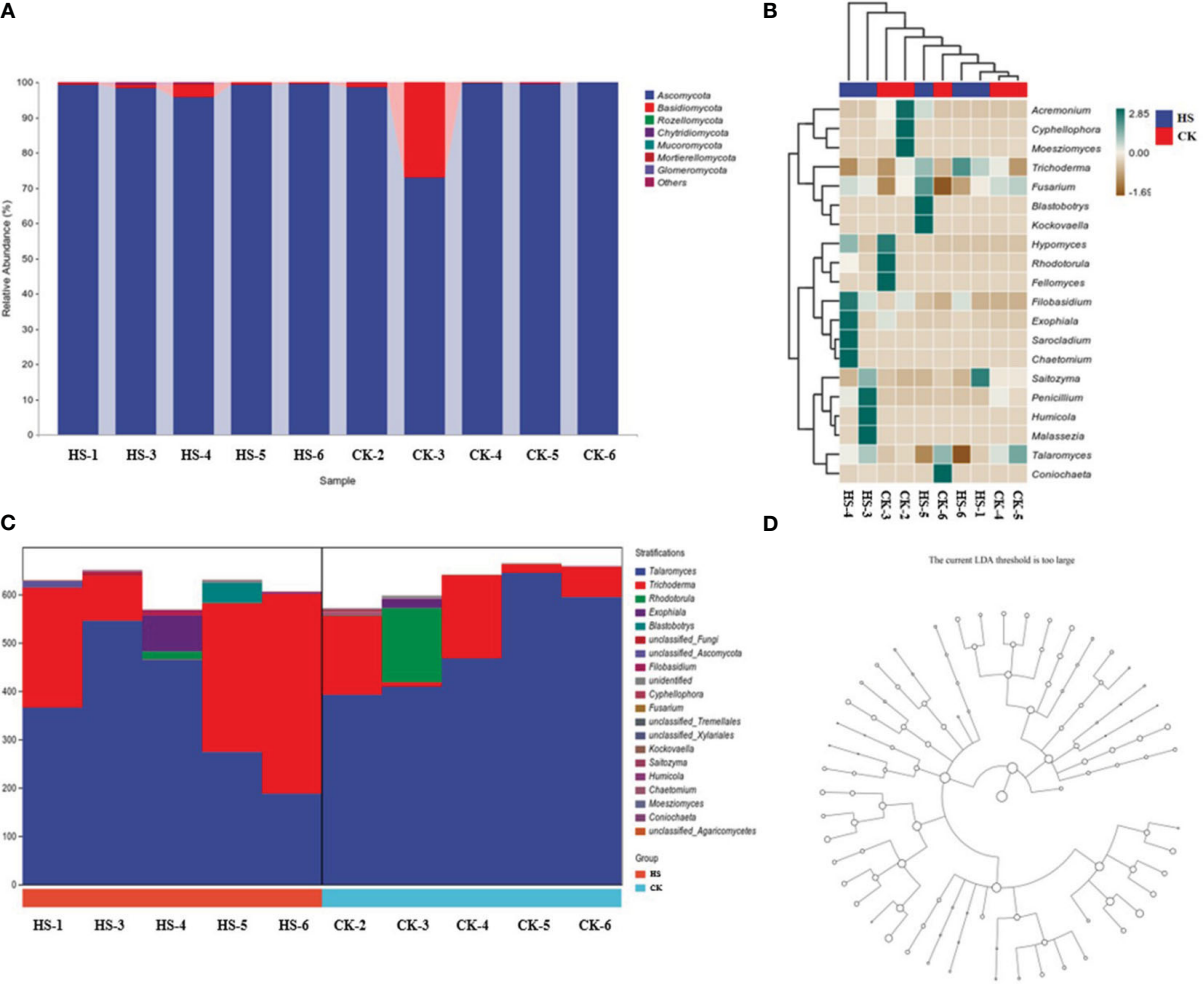
Comparison of endophytic fungal community structure in orchid roots under high temperature stress conditions. **(A)** Taxonomic composition analysis of fungi. **(B)** Heat map of fungal species composition. **(C)** Species composition diagram of fungal metabolic pathways. **(D)** Fungal LEfSe analysis. CK, control group; HS, high temperature stress; p: phylum, o: order, c: class, f: family, g: genus. The data in **Figures 4A, C** are respectively shown in **Supplementary Tables S2**, **S3**.

From **Figure 4A**, at the genus level, the relative abundance of endophytic fungi in orchid roots is dominated by *Ascomycota* (96.3%) and *Basidiomycota* (3.4%). Among them, compared to CK, the relative abundance of *Ascomycota* increased by 4.6% under HS, while the relative abundance of *Basidiomycota* decreased by 80.8%.

From **Figure 4B**, compared to CK, under HS, the community abundance of *Trichoderma*, *Blastobotrys*, *Kockovaella*, *Sarocladium*, and *Chaetomium* significantly increased, while the abundance of *Cyphellophora* and *Moesziomyces* decreased significantly.

From **Figure 4C**, under HS, compared to CK, at the genus level in the species composition of metabolic pathways, the relative abundance of endophytic fungi in orchid roots shows that *Talaromyces* decreased by 26.7%, *Trichoderma* increased by 150.6%, *Rhodotorula* decreased by 88.8%, and *Exophiala* increased by 291.4%.

From **Figure 4D**, at all levels, no population enrichment of endophytes fungal community structure in *P. aphrodite* roots.

### 3.5 Relationship between root endophytic microorganisms and their physiological indicators and negative oxygen ions concentration

**Figure 5:**
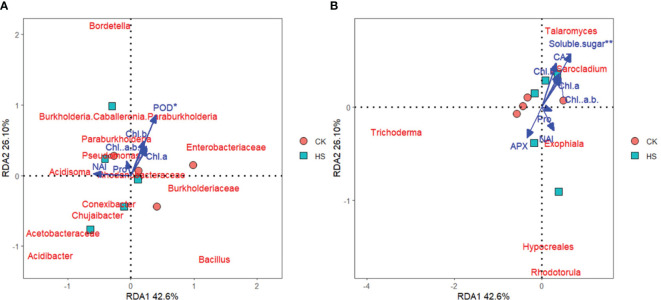
Redundancy analysis of physiological indicators and negative oxygen ions with root endophytic microbial community structure. **(A)** endophytic bacteria in roots; **(B)** endophytic fungi in roots. CK, control group; HS, high temperature stress treatment; NAI, Negative air ions; Chl a, Chlorophyll a; Chl b, Chlorophyll b; Chl a+b, Chlorophyll a+b; Pro, Proline; CAT, Catalase; POD, Peroxidase; APX, Ascorbate peroxidase.

As shown in **Figures 5A, B**, the community of root endophytic bacteria and fungi of *P. aphrodite* is significantly correlated with physiological indicators and negative oxygen ions concentration. Among them, POD has the greatest impact on the correlation between root endophytic bacteria. And there was an extremely significant correlation between Soluble sugar and root endophytes. NAI is significantly positively correlated with the abundance of root endophytic bacterial communities such as *Acetobacteraceae*, *Acidibacter*, *Chujaibacter*, *Acidisoma*, and with the abundance of root endophytic fungal communities such as *Exophiala*, *Rhodotorula* and *Hypocreales*.

## 4 Discussions

### 4.1 The impact of high temperature stress on the production of negative oxygen ions in *P. aphrodite*

The negative oxygen ions generating ability of *P. aphrodite* was significantly decreased under high temperature stress conditions. Under non-stress conditions, the highest concentration of negative oxygen ions typically occurs in the early evening (Li et al., 2022), which is consistent with the highest concentration of negative oxygen ions observed at 16:00 in this study. Previous research has shown that the intensity of photosynthesis in plants usually reaches its peak around 10:00 and then gradually decreases (Ma, 2022), which aligns with the phenomenon observed in this study that under HS conditions where the concentration of negative oxygen ions continuously rises before 12:00, peaks at 12:00, and then decreases. This indicates that the ability of plants to produce negative oxygen ions is influenced by the intensity of photosynthesis, increasing with higher photosynthetic activity. Shi et al. (2023) studied four forest parks in Beijing and found that as the environmental temperature rises, the concentration of negative oxygen ions in the air decreases. Additionally, due to various factors such as region, environment, and biology, there are differences in the concentration of negative oxygen ions at different times (Li et al., 2024).

### 4.2 The impact of high temperature stress on physiological indicators of *P. aphrodite*

High temperature stress affected the function of the osmoregulatory system of *P. aphrodite*, but the changes were not significant except for the significant increase of Pro content. The results of this experiment are consistent with Lee et al. (2021), indicating that high temperatures can induce stress responses in *P. aphrodite*. By enhancing the plant’s osmotic regulation capacity, reduce the reactive oxygen species content species to alleviate membrane oxidative damage, maintaining cell membrane permeability stability, the ability of *P. aphrodite* to resist high temperature stress could be enhanced. The dramatic changes in Pro content indicate that *P. aphrodite* plants are greatly affected by high temperature stress, and the physiological functions of the plants may be damaged.

High temperature stress activates the antioxidant system of *P. aphrodite*. Ko et al. (2020) found that under high temperature stress, the imbalance between the production and removal of reactive oxygen species leads to their excessive accumulation, which activates and increases the activity of POD and CAT, leading to its increased activity, which helps to maintain the stability of the plant body membrane system and thus resist the damage caused by high temperature stress. However, in this study, the activities of SOD and POD increased but not significantly, while the activities of CAT and APX decreased instead, which may be due to the high temperature of heat stress and destroyed the antioxidant system of *P. aphrodite*, thus leading to the abnormal performance of various antioxidant enzymes in *P. aphrodite* leaves (Chen, 2020). This indicates that *P. aphrodite* cannot adapt to the high temperature stress of 40°C.

Under high temperature stress conditions, the chlorophyll content of *P. aphrodite* increases and not significantly. Current research suggests that high temperature stress not only accelerates the degradation of chlorophyll but also inhibits its synthesis (Jiang et al., 2023), the results of this experiment are contrary to previous studies. It is speculated that the increased activity of POD in plants may play a reparative role, eliminating toxic substances accumulated under high temperature stress to ensure that *P. aphrodite* plants can maintain a relatively stable growth state under high temperature conditions (Wang, 1995; Zhang et al., 2017), thus leading to a slight increase in chlorophyll content in its leaves’ photosynthesis. As shown in **Figure 5A**, both POD content and chlorophyll content are positively correlated, so POD may have a role in promoting chlorophyll production.

### 4.3 Mechanism of changes in negative oxygen ions concentration under high temperature stress

The increase in chlorophyll content did not promote the production of more negative oxygen ions in *P. aphrodite*. On the contrary, under high temperature stress conditions, the concentration of negative oxygen ions produced by *P. aphrodite* was significantly lower than that of the control group and was not directly related to chlorophyll content (**Figure 5**). Previous studies have shown that chlorophyll is related to the photoelectric effect of plants and is an important natural photocatalyst for the production of negative oxygen ions in photosynthesis. A decrease in its content will reduce the photosynthetic capacity of plants, thereby weakening the ability of plants to produce negative oxygen ions. Research by Li et al. (2019) indicates that leaves generate negative oxygen ions through the photoelectric effect during photosynthesis, while plants release oxygen generated through photosynthesis into the air through stomata, providing a large amount of raw materials for the production of negative oxygen ions in the environment. Existing studies have found that high temperature stress has a strong inhibitory effect on the photosynthetic system function of plants, and PSII is the most sensitive part of the photosynthetic apparatus to high temperature stress. Therefore, it is speculated that high temperature stress leads to the destruction of the core protein components of PSII reaction centers in *P. aphrodite* seedlings, blocking the primary photosynthetic reactions, significantly reducing the efficiency of energy conversion, affecting electron transfer, and thus weakening the production of negative oxygen ions through the photoelectric effect of leaves. In addition, the inhibition of primary photosynthetic reactions may result in excess light energy, but plants may dissipate excess light energy through non-photochemical pathways, thereby alleviating damage from photooxidation to photosynthetic organs and chlorophyll, which may result in an increase in chlorophyll content. On the other hand, high temperature stress can induce stomatal closure in plants, reducing the concentration of oxygen released into the air through stomata, thereby reducing the raw materials needed for *P. aphrodite* to ionize the air and ultimately significantly reducing the production of negative oxygen ions.

### 4.4 The impact of high temperature stress on endophytic bacteria in the root system of *P. aphrodite* and its relationship with the physiological characteristics of *P. aphrodite* and the production of negative oxygen ions

The experimental results show that the abundances of the bacteria *Bacillus*, *Proteobacteria*, and *Pseudomonas* are significantly positively correlated with chlorophyll content. Bacillus significantly enriches under high temperature conditions, helping to maintain the internal microecological stability of the root of *P. aphrodite* (Lin et al., 2023). In addition, *Proteobacteria* and *Pseudomonas* both have roles in promoting the degradation and decomposition of straw and other materials in the soil (Leung et al., 2016; Fu et al., 2021; Zhang et al., 2022), which benefits *P. aphrodite* in absorbing mineral elements from soil, thereby synthesizing nutrients to produce chlorophyll, consistent with the slight increase in chlorophyll content observed in the results (**Figure 3D**). Moreover, redundancy analysis showed that chlorophyll content was positively related on the vertical axis, and thus the improvement of *Proteobacteria* and *Pseudomonas* richness may contribute to the improvement of chlorophyll content (**Figure 5A**).

The concentration of negative oxygen ions is positively correlated with the abundance of bacteria *Acetobacteraceae*, *Pseudomonas*, and *Acidobacteria* on the horizontal axis (**Figure 5A**). This may be due to the fact that some species of *Acetobacteraceae* have a photosynthesis-promoting effect (Mauro et al., 2023), which can help plants maintain the operation of the PSII reaction center, enhance photosynthesis and increase oxygen content, and ultimately benefit the photoelectric effect of ionizing oxygen to produce negative oxygen ions.

### 4.5 The impact of high temperature stress on the root endophytic fungi of *P. aphrodite* and its relationship with the physiological characteristics and the production of negative oxygen ions

High-temperature stress alters the structure of the endophytic fungal community in the root system of *P. aphrodite*. Compared to CK, under high-temperature stress, the abundance of *Trichoderma* and *Blastobotrys* communities increased. *Trichoderma* helps maintain soil health and is more conducive to the growth of *P. aphrodite* under high-temperature stress conditions (Shukla et al., 2022); *Blastobotrys* can effectively degrade cellulose (Boisramé and Neuvéglise, 2022), increasing the nutrient content available in the soil, which contributes to soil nutrient accumulation, meeting the nutrient supply requirements of *P. aphrodite* during stress, possibly related to the increase in their chlorophyll content. In addition, the abundance of the *Talaromyces* community decreased. *Talaromyces* has been shown to promote bioremediation (Assress et al., 2019), and the decrease in its abundance may not help *P. aphrodite* repair damage caused by high-temperature stress in adapting to high-temperature environments, reducing the ability of *P. aphrodite* to resist high-temperature stress. In this study, the abundance of *Rhodotorula* is positively correlated with the concentration of negative oxygen ions. *Rhodotorula* has antioxidant activity (Castañeda-Tamez et al., 2024), indicating that it may maintain membrane stability by clearing substances such as peroxides produced by *P. aphrodite* under high-temperature stress conditions, effectively alleviating PSII photoinhibition induced by high-temperature stress, and promoting the production of negative oxygen ions. However, due to the low abundance of *Rhodotorula* itself, its impact on the concentration of negative oxygen ions may be minimal.

### 4.6 Microorganisms mechanism of *P. aphrodite* regulating negative oxygen ions under high temperature stress

*P. aphrodite* resists high temperature stress through interactions with root endophytic microorganism. Root endophytic microorganisms can activate plant antioxidant enzymes, promote the synthesis of Pro, soluble sugars, and proteins in plants, enhance cellulose degradation to enrich the nutritional status of stressed plants, and help overcome high temperature stress (Shekhawat et al., 2022). We also found that endophytic bacteria in the roots play a dominant role in assisting plants in nutrient absorption, promoting soil organic matter decomposition, providing immunity against high temperature stress, and facilitating plant growth (Shaffique et al., 2022). Additionally, they indirectly enhance plant photosynthesis to increase the concentration of negative oxygen ions. In conclusion, root endophytic microorganisms play a crucial role in helping *P. aphrodite* cope with high temperature stress and maintaining its ability to release negative oxygen ions.

### 4.7 Mechanisms of P. aphrodite regulating negative oxygen ions release ability under high temperature stress

**Figure 6:**
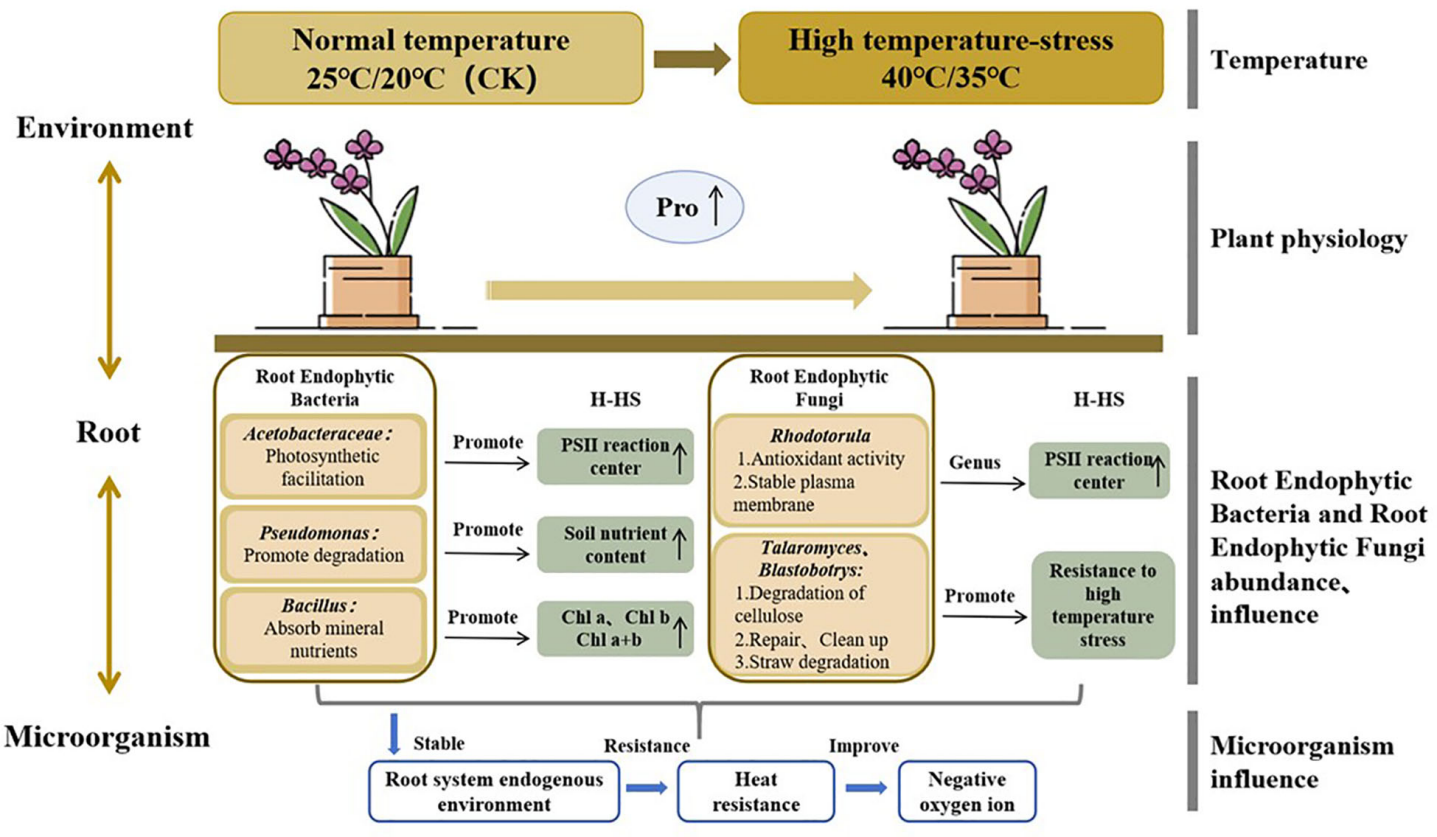
Conceptual framework of microbial regulatory mechanisms in response to high temperature stress in *P. aphrodite*.

As shown in **Figure 6**, high temperature has a profound impact on *P. aphrodite* and the community structure of root endophytic microorganisms. High temperature stress alters the abundance of endophytic bacteria such as Acetobacteraceae, Pseudomonas, Bacillus, Enterobacteriaceae (Sabir et al., 2020), and endophytic fungi such as Rhodotorula, Talaromyces, and Blastobotrys in the roots of *P. aphrodite*, which may favor the increased levels of Pro and chlorophyll in the leaves of *P. aphrodite*. This in turn enhances the resistance of *P. aphrodite* to high temperature stress, promoting the generation of negative oxygen ions. High temperature stress directly affects the physiological functions of *P. aphrodite*, including the osmotic regulation system and antioxidant system, and it is worth noting that there has been a significant increase in Pro content. While due to the significant impact of high temperature stress on *P. aphrodite* plants and the damage to their physiological functions, the supportive role of changes in microbial community structure is insufficient to fully offset the damage caused by high temperature stress, leading to a significant decrease in the ability of *P. aphrodite* to produce negative oxygen ions.

## 5 Conclusion

This study demonstrates that high temperature stress significantly reduces the ability of *P. aphrodite* to produce negative oxygen ions. Contrary to the common belief that higher chlorophyll content leads to stronger negative oxygen ions production, we speculate that high temperature stress disrupts the activity of PS II reaction centers, weakens plant photoelectric effects, and induces stomatal closure, reducing the oxygen content in the air, resulting in a significant decrease in negative oxygen ions production under high temperature stress conditions. Meanwhile, the Pro content significantly increases in the experiment, indicating a strong response of *P. aphrodite* to high temperature stress. Under high temperature stress conditions, the structure of the root endophytic microorganisms community in the roots undergoes significant changes, and the enrichment of beneficial microbial communities such as Enterobacteriaceae bacteria and Rhodotorula fungi helps increase Pro content, stabilize the endophytic environment of *P. aphrodite* roots, enhance the heat resistance of *P. aphrodite*, and their increased abundance contributes to the production of negative oxygen ions by *P. aphrodite*. However, the specific mechanism of their action requires further investigation.

## Data Availability

The original contributions presented in the study are publicly available. This data can be found at the National Center for Biotechnology Information (NCBI) using accession number PRJNA1143869.
