# Supplementary material for: Interactions between root endophytic microorganisms and the reduced negative ion release capacity of Phalaenopsis aphrodite Rchb. f. under high temperature stress
Source: Front Plant Sci. 2024 Aug 16;15:1437769. doi: 10.3389/fpls.2024.1437769 (PMC11361983; doi:10.3389/fpls.2024.1437769)
Supplement: Supplementary file 1 [file Table1.docx]

**Supplementary material**

**Interactions between root endophytic microorganisms and the reduced negative ion release capacity of** **Phalaenopsis aphrodite Rchb. f. under high temperature stress**

**Table S1.** Statistics of microbial taxa at each level of root endophytic bacterial community of Phalaenopsis aphrodite Rchb. f. (phylum level, HS-High temperature stress, CK-Control Check)

| Species | HS-1 | HS-3 | HS-4 | HS-5 | HS-6 | CK-2 | CK-3 | CK-4 | CK-5 | CK-6 |
| --- | --- | --- | --- | --- | --- | --- | --- | --- | --- | --- |
| Proteobacteria | 0.58530198 | 0.5473629 | 0.88772899 | 0.7011506 | 0.53847966 | 0.79750429 | 0.71816378 | 0.69690951 | 0.54128563 | 0.75541158 |
| Firmicutes | 0.2545147 | 0.24996064 | 0.08268364 | 0.05011967 | 0.34715598 | 0.00961377 | 0.03743679 | 0.14433572 | 0.13926273 | 0.00678078 |
| Actinobacteria | 0.07203066 | 0.10520861 | 0.0095261 | 0.10063609 | 0.05453983 | 0.10619024 | 0.15597303 | 0.08762837 | 0.16395553 | 0.13589008 |
| Acidobacteria | 0.03795897 | 0.06649173 | 0.0051895 | 0.0546632 | 0.04031973 | 0.04480405 | 0.04574248 | 0.02347736 | 0.07668645 | 0.0839636 |
| Chlamydiae | 0.00279612 | 0.000328 | 0.00591026 | 0.07654896 | 0.00030913 | 0.01740802 | 0.02546082 | 0.00014532 | 0.00208978 | 0.00319822 |
| Armatimonadetes | 0.02145507 | 0.0089609 | 0.00140549 | 0.00456913 | 0.00104516 | 0.00189695 | 0.00046218 | 0.00331008 | 0.00215665 | 0.00010981 |
| Bacteroidetes | 0.00159583 | 0.00136447 | 0.00092498 | 0.00061434 | 0.00206088 | 0.00056779 | 0.0006389 | 6.46E-05 | 0.00058514 | 0.0002608 |
| WPS-2 | 0.00073654 | 0.00162687 | 0.00010811 | 0.00071673 | 0.00017665 | 0.00086459 | 0.00165842 | 0.00119486 | 8.36E-05 | 0.00076867 |
| Patescibacteria | 0.00010912 | 0.00194175 | 6.01E-05 | 0.00011519 | 0 | 5.16E-05 | 0.00062531 | 0.00080734 | 0 | 5.49E-05 |
| Dependentiae | 0.00122756 | 0.00027552 | 0.00068473 | 0.00011519 | 0.00025025 | 0.00055489 | 0 | 0.00033908 | 0 | 0.00019217 |
| Others | 0.02227345 | 0.01647861 | 0.00577812 | 0.0107509 | 0.01566272 | 0.02054379 | 0.01383829 | 0.04178777 | 0.07389451 | 0.01336939 |

HS–The portion of microbes in different phyla in the endophytic bacterial community of Phalaenopsis roots under high temperature stress (40℃/35℃)，CK–The portion of microbes in different phyla in the endophytic bacterial community of Phalaenopsis roots under normal temperature conditions (25℃/20℃).

**Table S2.** Statistics of microbial taxa at each level of root endophytic fungal community of Phalaenopsis aphrodite Rchb. f. (phylum level, HS-High temperature stress, CK-Control Check)

| Species | HS-1 | HS-3 | HS-4 | HS-5 | HS-6 | CK-2 | CK-3 | CK-4 | CK-5 | CK-6 |
| --- | --- | --- | --- | --- | --- | --- | --- | --- | --- | --- |
| Ascomycota | 0.99618928 | 0.97233211 | 0.99779995 | 0.8268148 | 0.98468867 | 0.94380825 | 0.99880283 | 0.98427724 | 0.99357956 | 0.99295862 |
| Basidiomycota | 0.00306847 | 0.02693422 | 0.00141623 | 0.07934319 | 0.01203756 | 0.04871475 | 0.00025588 | 0.00112714 | 0.00419426 | 0.00254272 |
| Rozellomycota | 1.53E-05 | 0 | 5.34E-05 | 3.56E-05 | 0 | 1.52E-05 | 0 | 0 | 0 | 0.00068973 |
| Mucoromycota | 0 | 0 | 0 | 0 | 0 | 0 | 0 | 0 | 0 | 0 |
| Glomeromycota | 0 | 0 | 0 | 0 | 0 | 0 | 0 | 0 | 0 | 0 |
| Chytridiomycota | 0 | 0 | 0 | 0 | 0 | 0 | 0 | 0 | 0 | 0 |
| Mortierellomycota | 0 | 0 | 0 | 0 | 0 | 0 | 0 | 0 | 0 | 0 |
| Others | 0.00072694 | 0.00073368 | 0.00073038 | 0.09380643 | 0.00327377 | 0.00746178 | 0.00094128 | 0.01459562 | 0.00222618 | 0.00380894 |

HS–The portion of microbes in different phyla in the endophytic bacterial community of Phalaenopsis roots under high temperature stress (40℃/35℃)，CK–The portion of microbes in different phyla in the endophytic bacterial community of Phalaenopsis roots under normal temperature conditions (25℃/20℃).

**Table S3.** Species composition statistics of metabolic pathways of root endophytic fungi from Phalaenopsis aphrodite Rchb. f. (genus level, HS-High temperature stress, CK-Control Check)

| Species | HS-1 | HS-3 | HS-4 | HS-5 | HS-6 | CK-2 | CK-3 | CK-4 | CK-5 | CK-6 |
| --- | --- | --- | --- | --- | --- | --- | --- | --- | --- | --- |
| Talaromyces | 366.128142 | 545.669315 | 465.011173 | 274.453246 | 188.160923 | 392.702466 | 409.226445 | 467.753693 | 644.731751 | 594.734648 |
| Trichoderma | 246.531993 | 92.116557 | 0.52061 | 307.461167 | 412.654942 | 163.130807 | 8.913744 | 171.725798 | 17.016303 | 61.891863 |
| Rhodotorula | 0.015075 | 0 | 16.548017 | 0.803595 | 0.063303 | 1.677106 | 153.920613 | 0.399502 | 0.053482 | 0.062186 |
| Exophiala | 0.271342 | 0.518648 | 75.001574 | 0.105579 | 2.258779 | 1.532228 | 18.086193 | 0 | 0.051478 | 0.298821 |
| Blastobotrys | 0 | 0 | 0 | 41.685273 | 0 | 0 | 0 | 0.093307 | 0 | 0 |
| unclassified_Fungi | 1.317111 | 6.16736 | 3.958996 | 0.267054 | 0.419967 | 1.955006 | 0.375811 | 0.285454 | 0.724132 | 0.406312 |
| unclassified_Ascomycota | 13.482679 | 0.458593 | 0.012545 | 0.026395 | 0 | 0 | 0.103711 | 0.013219 | 0 | 0 |
| Filobasidium | 0.042564 | 1.888965 | 5.850183 | 0.304317 | 1.677524 | 1.763842 | 0.653866 | 0.037323 | 0.046331 | 0 |
| unidentified | 0.136854 | 1.096214 | 0.011807 | 0.547392 | 0.194012 | 0.914586 | 3.126081 | 0.098146 | 0.538596 | 0.038268 |
| Cyphellophora | 0 | 0 | 0 | 0.052791 | 0 | 5.590831 | 0.41947 | 0 | 0.021879 | 0 |
| Fusarium | 0.457558 | 0.518648 | 0.602137 | 0.801161 | 0.305963 | 0.486988 | 0.314596 | 0.559843 | 0.62289 | 0.187397 |
| unclassified_Tremellales | 0 | 0 | 0 | 0 | 0 | 0.659215 | 2.00478 | 0.024882 | 0.453011 | 0.01013 |
| unclassified_Xylariales | 0 | 0 | 0 | 1.99359 | 0 | 0 | 0 | 0 | 0 | 0 |
| Kockovaella | 0 | 0.016378 | 0 | 1.739652 | 0 | 0 | 0 | 0 | 0.020591 | 0 |
| Saitozyma | 0.758771 | 0.500157 | 0 | 0 | 0 | 0 | 0.040782 | 0.135126 | 0.140997 | 0.062186 |
| Humicola | 0 | 1.430376 | 0 | 0 | 0 | 0 | 0 | 0 | 0 | 0 |
| Chaetomium | 0 | 0 | 1.239697 | 0 | 0 | 0 | 0 | 0 | 0 | 0 |
| Moesziomyces | 0 | 0 | 0 | 0 | 0 | 1.092753 | 0 | 0 | 0.015444 | 0 |
| Coniochaeta | 0 | 0.021838 | 0 | 0 | 0 | 0 | 0 | 0 | 0 | 1.04841 |
| unclassified_Agaricomycetes | 0.668321 | 0.144375 | 0 | 0 | 0 | 0.083144 | 0 | 0 | 0 | 0 |

HS–The portion of microbes in different phyla in the endophytic bacterial community of Phalaenopsis roots under high temperature stress (40℃/35℃)，CK–The portion of microbes in different phyla in the endophytic bacterial community of Phalaenopsis roots under normal temperature conditions (25℃/20℃).
